# Unmasking the Clock: Diurnal Blood Pressure Variation in Patients With Type 2 Diabetes Mellitus With Hypertension via Ambulatory Blood Pressure Monitoring (ABPM)

**DOI:** 10.7759/cureus.94968

**Published:** 2025-10-20

**Authors:** Shiv Shankar, Ramesh Aggarwal, Anupam Prakash, Sameer Gulati, Shubhalaxmi Margekar, Tushar Shailat

**Affiliations:** 1 Internal Medicine, Lady Hardinge Medical College, New Delhi, IND

**Keywords:** ambulatory blood pressure monitoring (abpm), diurnal rhythm, hypertension, nondipping, reverse dipping, type 2 diabetes mellitus (t2dm)

## Abstract

Introduction

Hypertension and type 2 diabetes mellitus (T2DM) frequently coexist, significantly increasing cardiovascular risk. The diurnal variation in blood pressure (BP) is well-established, with BP typically dipping during sleep. However, in T2DM patients with hypertension, this rhythm is often disrupted. This study aims to assess diurnal BP variations in such patients using ambulatory blood pressure monitoring (ABPM).

Methods

This descriptive cross-sectional study included 100 T2DM patients with hypertension from the Department of Medicine at Lady Hardinge Medical College, New Delhi. ABPM was performed on all participants, recording BP every 30 minutes during the day and every 60 minutes at night. Patients were categorized into normal dippers, nondippers, and reverse dippers based on their nocturnal BP patterns. Statistical analyses, including Chi-square tests and Wilcoxon-Mann-Whitney U tests, were used to determine associations between diurnal rhythm loss and clinical parameters.

Results

The study found that 88% of participants exhibited a loss of diurnal BP rhythm, with 57% categorized as nondippers and 31% as reverse dippers. Only 12% of patients maintained a normal dipping BP pattern. The findings suggest a higher prevalence of diurnal rhythm loss among female patients (93%) compared to males (75%) (p = 0.034). There was no significant association between BP control and diurnal rhythm loss (p = 0.527), but patients taking antihypertensive medication both in the morning and evening had a significantly lower incidence of diurnal rhythm loss compared to those taking medication only in the morning (p < 0.001).

Conclusion

This study highlights that the majority of T2DM patients with hypertension exhibit abnormal BP patterns, which may predispose them to an increased risk of cardiovascular complications. Ambulatory BP monitoring is essential for detecting these patterns and optimizing treatment strategies. The findings emphasize the importance of individualized antihypertensive therapy, particularly with a focus on medication timing, to improve diurnal BP rhythm. Future studies should explore larger sample sizes and long-term follow-ups to further validate these findings and assess the impact of chronotherapy on clinical outcomes.

## Introduction

Hypertension and type 2 diabetes mellitus (T2DM) frequently coexist, leading to a significantly increased risk of cardiovascular disease, chronic kidney disease, and cerebrovascular complications [1]. Additionally, insulin resistance promotes weight gain and visceral fat accumulation, triggering chronic inflammation [2]. One of the key physiological processes regulating blood pressure (BP) is its diurnal variation, with BP typically dipping during nighttime sleep. This circadian BP rhythm is influenced by autonomic nervous system activity, hormonal fluctuations, and vascular tone [3-6]. However, in individuals with T2DM and hypertension, this physiological dip is often blunted or completely absent, resulting in nondipping or reverse-dipping patterns [7-9].

The absence of a normal nocturnal dip has been associated with increased cardiovascular morbidity and mortality, warranting a closer investigation of BP patterns in this population [9]. Ambulatory blood pressure monitoring (ABPM) offers a more comprehensive assessment of BP fluctuations over a 24-hour period, allowing for the identification of these abnormal BP variations. This study aims to analyze the prevalence of nondipping and reverse-dipping patterns in T2DM patients with hypertension and to evaluate potential associations with clinical parameters such as gender, BMI, and medication timing.

Circadian rhythms and BP

Circadian rhythms are 24-hour oscillations crucial for many physiological functions, including BP regulation. The body’s central clock, located in the suprachiasmatic nucleus (SCN) of the hypothalamus, along with peripheral clocks in organs like the kidney and liver, plays a vital role in controlling BP fluctuations throughout the day [10]. BP follows a circadian pattern, typically characterized by higher BP during the day and a dip at night. This phenomenon, referred to as the nocturnal dip, usually results in a 10-20% reduction in BP during sleep [11]. The natural circadian rhythm is influenced by intrinsic factors such as neurohormonal regulation and external factors like physical activity, sodium intake, and emotional stress [12]. 

Types of circadian variation of BP: dippers and nondippers

Dippers are individuals whose BP decreases by more than 10% during sleep compared to daytime BP. Nondippers show less than a 10% reduction or no reduction in BP during the night [13]. Nondipping status is associated with higher cardiovascular morbidity and mortality, including increased risks of stroke, myocardial infarction, and progression of renal disease [14].

Pathophysiological Mechanisms Linked to Circadian BP Variation

Renin-angiotensin-aldosterone system (RAAS): This system shows diurnal variation, with plasma renin activity peaking in the early morning, which may contribute to the morning BP surge [15].

Catecholamines (epinephrine and norepinephrine): These hormones exhibit a similar circadian pattern, with levels rising in the early morning and contributing to increased vascular resistance and BP upon awakening [16].

Hematologic factors: Blood viscosity and platelet aggregation increase in the morning, which, along with reduced plasminogen activator activity, contribute to the higher risk of thrombosis and cardiovascular events in the morning [17-18].

Sympathetic nervous system (SNS): The SNS plays a significant role in regulating BP, especially during sleep and upon waking. An early morning rise in BP, driven by sympathetic nervous system activation, is associated with increased heart rate, vascular resistance, and decreased vagal activity [12].

Salt sensitivity: Sodium intake can significantly influence the circadian rhythm of BP. In sodium-sensitive hypertensive patients, salt restriction has been shown to shift the circadian rhythm from a nondipper to a dipper pattern [19].

Extrinsic Factors Influencing Circadian BP

Sleep quality and activity: Poor sleep or physical inactivity can disrupt circadian BP rhythms. Sleep deprivation increases sympathetic activity, leading to higher BP during the night [20-21].

Dietary factors: Sodium and potassium intake influence BP rhythms. Salt loading can enhance the morning BP surge of 51 mmHg, while potassium supplementation has been shown to convert nondippers to dippers in some cases [22].

ABPM for Hypertension Diagnosis

The international guidelines from North America, Europe, Japan, and China, as well as the 2017 American College of Cardiology (ACC)/American Heart Association (AHA) guidelines, agree that ABPM is the gold standard for diagnosing hypertension. ABPM helps confirm that an elevated BP in the clinic is consistent and not due to a temporary white-coat effect (which occurs in about 25% of patients). ABPM should be offered to anyone with elevated BP in any setting, including at the clinic, home, pharmacy, or kiosk, to confirm the diagnosis of hypertension and to guide treatment decisions [23].

ABPM for Evaluating Treatment Efficacy

ABPM is effective in guiding the prescription of hypertension medications. In one study, only 12% of patients reached their BP target based on office readings, but with ABPM, one-third of patients met their BP goals. In many cases, ABPM resulted in changes to medication that improved BP control. ABPM is also cost-effective, as it helps avoid unnecessary treatments for patients with white-coat hypertension and ensures that patients with masked hypertension receive appropriate medication [24].

Long-Term Monitoring and Control With ABPM

Once BP control is achieved, ABPM should be performed periodically to ensure 24-hour control. Home BP monitoring can complement ABPM for daytime readings, but ABPM remains essential for tracking nocturnal BP [25].

Research findings and recommendations

Studies suggest that ABPM-guided management of hypertension, combined with chronotherapy, leads to better outcomes in diabetic patients. For example, in the Hygia Project, hypertensive patients with diabetes who took their antihypertensive medication at bedtime had better BP control, reduced target organ damage, and lower rates of cardiovascular events [26].

ABPM is essential for managing hypertension in diabetic patients because it allows for a comprehensive assessment of 24-hour BP patterns, particularly during sleep. The identification of nocturnal hypertension and abnormal circadian BP rhythms enables more precise treatment plans aimed at reducing cardiovascular events [26].

Combining ABPM with chronotherapy (bedtime medication) has been shown to significantly improve BP control, especially nocturnal BP, and reduce cardiovascular risk in diabetic patients. This approach should be considered the preferred strategy for treating hypertensive patients with diabetes [26].

## Materials and methods

The study was approved by the institutional ethics committee of Lady Hardinge Medical College & SSK Hospital with the approval number LHMC/IEC/2023/PG Thesis/45. A descriptive cross-sectional study was conducted in a tertiary care hospital in India. A convenient sample size of 100 patients was taken over a 19-month period from May 2023 to November 2024. Patients from the outpatient department (OPD) of general medicine and the diabetes clinic of Lady Hardinge Medical College and SSKH, New Delhi, were evaluated for inclusion and exclusion criteria. Inclusion criteria included patients more than or equal to 18 years of age, those with T2DM with hypertension as defined by the American Diabetes Association (ADA) 2022 and ACC/AHA 2017 guidelines, respectively, and those on a stable dose of medication for both of these conditions for at least four weeks. The exclusion criteria included patients requiring urgent modification of therapy for T2DM and hypertension, bedridden or moribund patients, pregnant or lactating females, and individuals with chronic kidney disease (CKD) Stage III or above. Information obtained from clinical examination and available records of the patient was used for evaluation of the inclusion and exclusion criteria. Patients who fulfilled all the inclusion criteria and none of the exclusion criteria were included in the study and were given the patient information sheet and the informed consent form in the language of their understanding, and details of the study were explained. Once the subjects agreed to participate in the study, written informed consent was obtained from them or their legally acceptable representatives.

A complete general physical examination was performed for each subject enrolled in the study. The collected information was noted on a prestructured, pretested proforma. As part of the study, the subjects wore a portable programmable ABPM device (Model BR 102PLUS, No.293.09921) that was programmed to automatically measure the BP every 30 minutes during the day and every 60 minutes during the night for a period of 24 hours.

**Figure 1:**
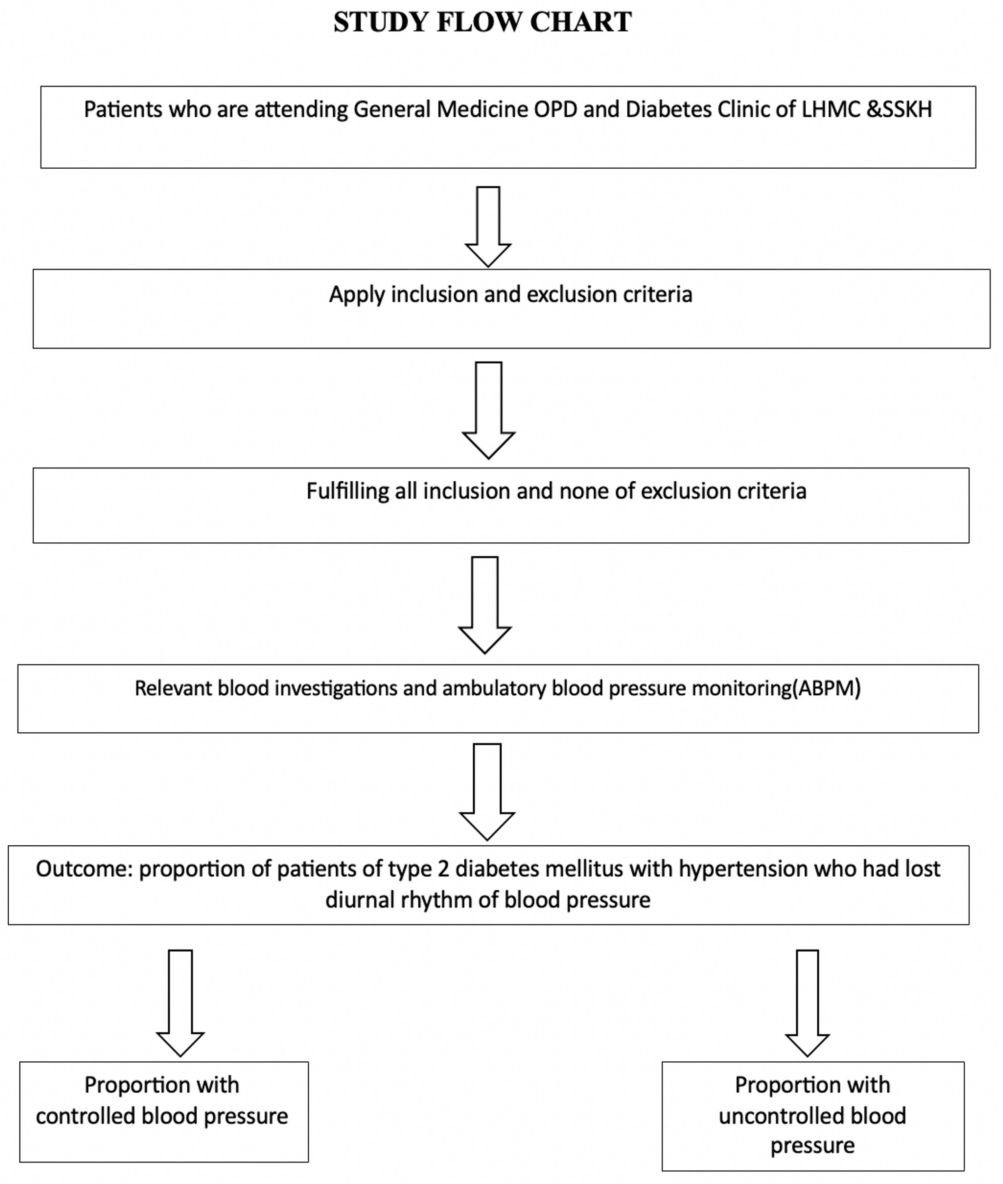
Flowchart depicting methodological recruitment and allocation to subgroups OPD: outpatient department; LHMC: Lady Hardinge Medical College; SSKH: Shrimati Sucheta Kriplani Hospital

At the time of placement, ambulatory BP reading was cross-checked with manual BP readings. An ambulatory BP apparatus cuff was tied on the nondominant arm of the subjects, and the subjects were asked to follow their usual daily routine activities. Subjects were taught to tie the cuff, just in case it appeared to be too loose or too tight for the patient at home. A flowchart depicting the methodological recruitment and allocation into subgroups is shown in Figure 1.

**Figure 2:**
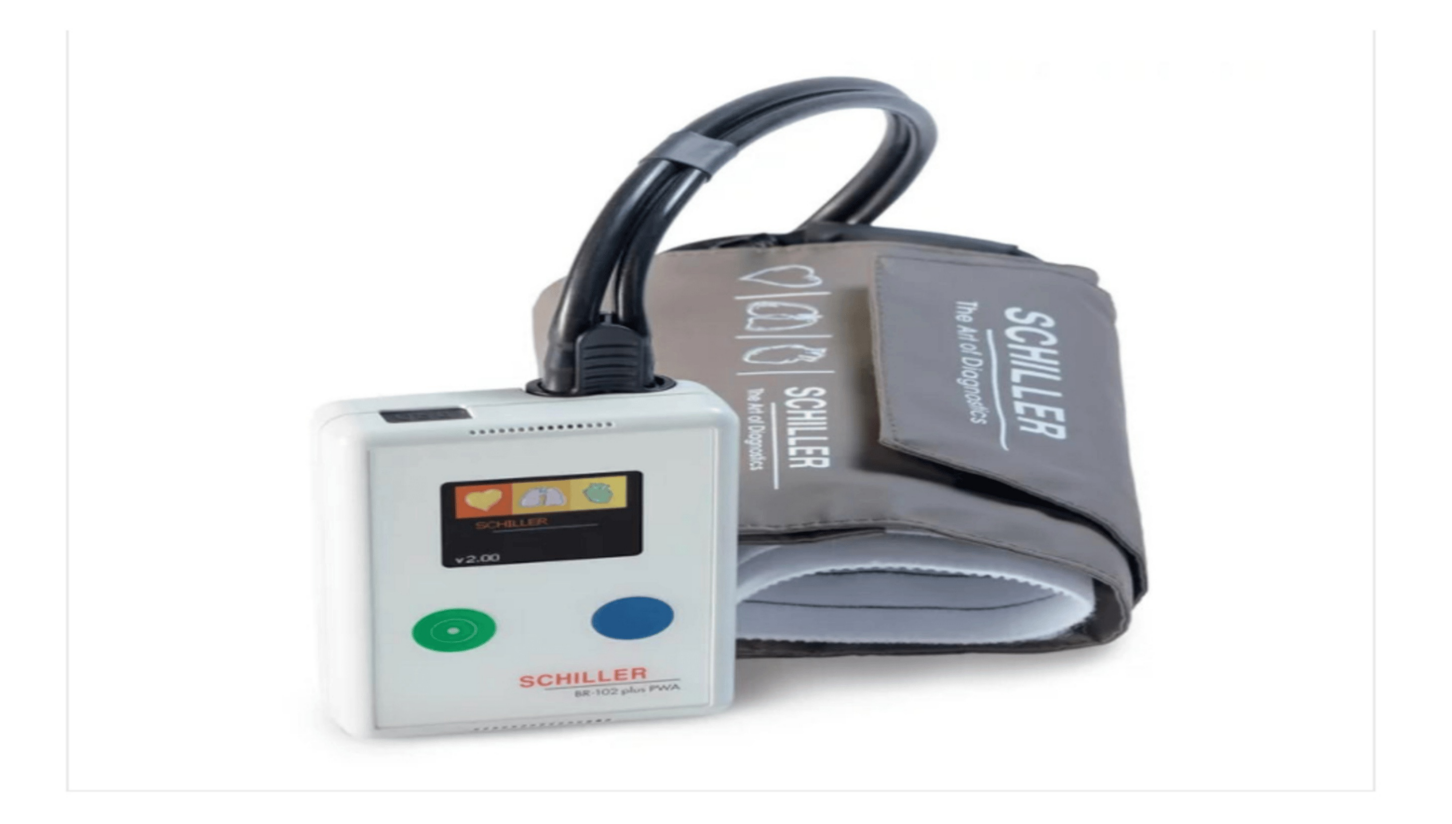
SCHILLER BR-102 (CE-certified) ambulatory blood pressure monitoring device CE:  Conformité Européenne Image of ambulatory blood pressure monitor; created by authors; no third-party permission required

A SCHILLER BR-102 (CE-certified) ABPM device used in the study is shown in Figure 2.

Once the device was removed from the subjects after 24 hours of readings, all recorded readings were obtained by attaching them to a computer with preloaded software. It provided the average of 24 hours, awake time, and sleep time readings, as well as a graph of the subject’s BP level during 24 hours. A copy of the readings obtained was provided to the subjects. Though not a part of the research but to benefit the participants, necessary therapeutic advice was provided to the participants after evaluating the ambulatory BP readings. Daytime systolic BP (SBP)/diastolic BP (DBP), nighttime SBP/DBP, and Diurnal index (DI) readings were recorded. Collected data was transferred to an MS Excel (Microsoft Corporation, Redmond, Washington, United States) spreadsheet.

Outcome Variables

Primary outcome variable: Proportion of patients with T2DM with hypertension who had lost the diurnal rhythm of BP on ABPM.

Secondary outcome variable: The proportion of patients who had lost the diurnal rhythm of BP in patients with controlled BP compared to those who had uncontrolled BP.

Statistical analysis

The data was entered in an MS spreadsheet, and analysis was done using IBM SPSS Statistics for Windows, Version 31 (Released 2025; IBM Corp., Armonk, New York, United States). Descriptive statistics were used to summarize the demographic and clinical characteristics of the study population. The Chi-square test was used to assess associations between categorical variables, while the Wilcoxon-Mann-Whitney U test was used for nonparametric comparisons. A p-value of <0.05 was considered statistically significant.

## Results

A total of 100 subjects were enrolled in the study to assess the proportion of patients with T2DM with hypertension who had lost diurnal rhythm on ABPM. All subjects included were 18 years and older, with a mean age of 55.00 ± 9.96 years. The median age of the population was also 55 years, with an interquartile range (IQR) of 49-61 years. The youngest participant was 31 years old, while the oldest was 81 years old, illustrating a wide range of ages in the study. In our study, 72 patients were females, while 28 patients were males. Table 1 shows baseline characteristics of study participants.

**Table 1:** Baseline characteristics of participants M: male; F: female; BMI: body mass index; BP: blood pressure; HBA1C: hemoglobin A1C Data presented as mean ± standard deviation, absolute numbers

Parameter	
Mean age (years)	55 ± 9.96
Gender (M/F)	28/72
Mean BMI (kg/m^2^)	24.00 ± 1.98
Mean systolic BP (mm/Hg)	125.81 ± 14.75
Mean HBA1C (gm/dl)	7.01 ± 0.62
Duration of diabetes (years)	12.29 ± 5.65
Duration of hypertension (years)	10.69 ± 4.66

The study comprehensively evaluated the diurnal rhythm of BP in patients with T2DM and hypertension using ABPM. The findings highlighted the prevalence of abnormal BP patterns, with 88% of participants losing their diurnal rhythm, defined by either a nondipping or reverse-dipping BP pattern. Table 2 shows the diurnal rhythm variation of study participants.

**Table 2:** Diurnal rhythm variation of study participants Data presented as absolute numbers, percentage

Diurnal rhythm	Frequency (percentage)	
Lost	88 (88.0%)	
Maintained	12 (12.0%)	
Total	100 (100%)	

Among the participants, 57% demonstrated a nondipping pattern (no significant drop in nighttime BP compared to daytime levels), and 31% exhibited a reverse-dipping pattern, where nighttime BP exceeded daytime BP. Only 12% maintained a normal dipping BP pattern. Table 3 shows the BP pattern of the study participants. 

**Table 3:** Blood pressure pattern of the study participants Data presented as absolute numbers, percentage

Blood pressure pattern	Frequency (percentage)	
Normal dipping	12 (12.0%)	
Nondipping	57 (57.0%)	
Reverse dipping	31 (31.0%)	
Total	100 (100%)	

No significant association was found between BP control and diurnal rhythm loss, with 60.2% of participants with controlled BP and 39.8% with uncontrolled BP demonstrating rhythm loss (p = 0.527). This finding suggests that circadian BP dysregulation occurs independently of BP control. Table 4 shows the association between diurnal rhythm and BP control. 

**Table 4:** Association between diurnal rhythm and blood pressure control Data presented as absolute numbers, percentage

Blood pressure	Diurnal rhythm			Fisher's Exact Test	
	Lost	Maintained	χ2		p-value
Controlled	53 (60.2%)	9 (75.0%)	0.978		0.527
Not controlled	35 (39.8%)	3 (25.0%)			
Total	88 (100.0%)	12 (100.0%)			

The study evaluated the association between diurnal rhythm and the timing of antihypertensive medication intake (morning only vs. morning + evening). The results demonstrated a statistically significant association between drug timing and the likelihood of maintaining or losing the diurnal rhythm (p = 0.0006). Among participants who lost their diurnal rhythm, 77.3% took their medication in the morning only, while 22.7% followed a morning and evening regimen. Conversely, among those who maintained their diurnal rhythm, 25% took their medication in the morning only, and 75% adhered to a morning + evening regimen. Table 5 shows the association between the diurnal rhythm and the timing of drug intake.

**Table 5:** Association between diurnal rhythm and timing of drug intake Data presented as absolute numbers, percentages

Diurnal rhythm	Drug timing		Fisher's Exact Test	
	Morning	Morning + evening	χ2	p-value
Lost	68 (95.8%)	20 (69.0%)	14.014	<0.001
Maintained	3 (4.2%)	9 (31.0%)		
Total	71 (100.0%)	29 (100.0%)		

The mean duration of hypertension in this study was 10.69 years, with a significantly longer duration observed in participants who lost their diurnal rhythm (mean, 11.15 years) compared to those who maintained it (mean, 7.33 years; p = 0.0080), which demonstrated that increasing age and prolonged hypertension are associated with a higher prevalence of nondipping BP patterns in diabetic patients. Table 6 shows the association between the diurnal rhythm and the duration of hypertension (years).

**Table 6:** Association between the diurnal rhythm and the duration of hypertension SD: standard deviation; IQR: interquartile range Data presented as absolute numbers, percentage

Duration of hypertension (years)	Diurnal rhythm		Wilcoxon-Mann-Whitney U Test	
	Lost	Maintained	W	p-value
Mean (SD)	11.15 (4.61)	7.33 (3.73)	778.500	0.008
Median (IQR)	11 (8-14.25)	7.5 (3.75-10)		
Min-max	2-23	3-15		

In our study, the mean duration of diabetes in the participants was 12.29 years, with no significant difference between participants who maintained their diurnal rhythm and those who lost it (p = 0.126). Table 7 shows the association between the diurnal rhythm and the duration of diabetes (years).

**Table 7:** Association between diurnal rhythm and duration of diabetes SD: standard deviation; IQR: interquartile range Data presented as absolute numbers, percentages

Duration of diabetes (years)	Diurnal rhythm		Wilcoxon-Mann-Whitney U Test	
	Lost	Maintained	W	p-value
Mean (SD)	12.58 (5.56)	10.17 (6.10)	672.500	0.126
Median (IQR)	13 (8-15.25)	8 (6.25-12.5)		
Min-max	2 - 32	3 - 22		

## Discussion

This study provides a comprehensive examination of circadian BP regulation in patients with T2DM and concomitant hypertension, utilizing 24-hour ABPM to characterize diurnal BP patterns. Our findings reveal a remarkably high prevalence (88%) of abnormal circadian BP profiles in this population, with 57% exhibiting a nondipping pattern (nocturnal BP reduction <10%) and 31% demonstrating the more pathological reverse-dipping pattern (nocturnal BP exceeding daytime values). These results substantially exceed previously reported rates in comparable populations, suggesting that our cohort may represent a distinct clinical phenotype with particularly severe circadian BP dysregulation.

The observed gender disparity in circadian BP disruption merits careful consideration. While epidemiological studies typically report worse nocturnal BP control in males [27], our findings demonstrate a significantly higher prevalence of rhythm loss in female participants (93% vs. 75% in males, p = 0.034). This unexpected result may reflect several potential mechanisms: (1) the loss of estrogen-mediated vascular protection in postmenopausal women, as estrogen is known to enhance endothelial function and arterial compliance [1]; (2) gender differences in autonomic nervous system regulation of cardiovascular function; or (3) variations in body composition and fat distribution that may differentially affect nocturnal hemodynamics. The predominance of female participants (72%) in our study cohort suggests the need for gender-stratified analyses in future investigations of circadian BP regulation.

The strong association between hypertension duration and circadian rhythm disruption (11.15 vs. 7.33 years in rhythm-maintained group, p = 0.008) supports the concept of progressive vascular damage impairing normal BP variation. Chronic pressure overload leads to structural changes in resistance vessels, including medial hypertrophy and reduced vascular compliance, which may blunt the normal nocturnal BP decline [28]. Additionally, long-standing hypertension is associated with baroreflex dysfunction and increased sympathetic nervous system activity, both of which can disrupt circadian BP patterns. The trend toward longer diabetes duration in rhythm-loss patients (12.58 vs. 10.17 years, p = 0.126) suggests that chronic hyperglycemia may contribute to circadian disruption through multiple pathways, including advanced glycation end-product accumulation, oxidative stress, and autonomic neuropathy, though these relationships require further elucidation.

Therapeutic implications emerge from our finding that medication timing significantly influenced rhythm preservation. Patients receiving antihypertensive therapy in both morning and evening demonstrated superior circadian BP profiles compared to those on once-daily morning regimens (p < 0.001). This observation aligns with the chronotherapeutic principle that drug administration timing should align with circadian patterns of disease pathophysiology [29]. The renin-angiotensin-aldosterone system exhibits diurnal variation in activity, peaking in the early morning hours, which may explain the particular efficacy of evening-dosed RAAS inhibitors in restoring nocturnal BP dipping. Our results suggest that even simple twice-daily dosing strategies, without requiring precise bedtime administration, may offer meaningful improvements in circadian BP regulation.

From a clinical perspective, these findings highlight the critical importance of ABPM in the comprehensive evaluation of diabetic hypertensives. Conventional office BP measurements fail to capture these circadian abnormalities, potentially missing an important dimension of cardiovascular risk assessment. The high prevalence of reverse-dipping (31%) in our cohort is particularly concerning, as this pattern has been associated with the worst cardiovascular outcomes among all BP phenotypes [30]. Our results suggest that female gender and longer hypertension duration may serve as clinical markers identifying patients who would benefit most from ABPM evaluation and subsequent chronotherapeutic interventions.

Limitations

This study has several limitations. The small sample size (n = 100) and its conduct within a single tertiary care hospital limit broader applicability, as the patient population may not reflect the general population of individuals with T2DM and hypertension. A higher proportion of female participants (72%) may also skew results, reducing their relevance to male patients. Additionally, psychological factors and sleep disorders, which can significantly influence BP rhythms, were not evaluated. The potential discomfort or sleep disruption caused by ABPM devices could have impacted nighttime readings. Moreover, while the study explored drug timing, it did not account for specific classes or combinations of antihypertensive and antidiabetic medications that may affect BP variability. Finally, the exclusion of patients with advanced chronic kidney disease, pregnant or lactating women, and those requiring urgent therapy adjustments may have led to the omission of key subgroups with potentially different BP profiles.

## Conclusions

This study demonstrated that the majority of T2DM patients with hypertension exhibit an altered diurnal BP pattern, with a high prevalence of nondipping and reverse-dipping phenotypes. These abnormal BP variations have been strongly associated with increased cardiovascular risk. The findings underscore the importance of ABPM in detecting and managing these BP patterns effectively. The study also highlights the role of medication timing in maintaining BP rhythms, suggesting that antihypertensive chronotherapy may be beneficial. Future studies with larger sample sizes and longer follow-up durations are warranted to validate these findings and explore additional therapeutic strategies for optimising BP control in diabetic hypertensive patients.
